# Dry Spin Graphene Oxide Fibers: Mechanical/Electrical Properties and Microstructure Evolution

**DOI:** 10.1038/s41598-018-29157-4

**Published:** 2018-07-17

**Authors:** Lichao Feng, Ying Chang, Jing Zhong, De-Chang Jia

**Affiliations:** 1 School of Mechanical and Ocean Engineering, Huaihai Institute of Technology, and Marine Resources Development Institute of Jiangsu, Lianyungang, 222005 Jiangsu China; 2 Key Lab of Structure Dynamic Behavior and Control (Harbin Institute of Technology), Ministry of Education, Harbin, 150090 Heilongjiang China; 3 School of Civil Engineering, Harbin Institute of Technology, Harbin, 150090 China; 4 Institute for Advanced Ceramics, Harbin Institute of Technology, Harbin, 150080 China; 5 School of Materials Science and Engineering, Harbin Institute of Technology, Harbin, 150010 China

## Abstract

Dry-spinning method is extensively employed in fiber industry, comparing to the counter-part of wet-spinning process, it has advantages of environmentally friendly, high yield rate and no need for purification. Here, we report the synthesis of graphene oxide (GO) fibers via dry spinning GO inks with extremely high concentrations. The proper rheology properties of such GO inks allow us to dry spin GO fiber directly. Various dry spinning conditions are investigated, and the relationship between mechanical performance and micro-structure of the obtained GO fiber are established. We found that the existence of larger GO liquid crystal domains does not necessarily result to higher mechanical properties, and it is because those large GO liquid crystal domains evolve into thick GO films during drying process and thus prevent the intimate compaction of the whole GOF and leave behind gaps. This is detrimental for the mechanical properties, and thus the dry spin GOF are much weaker than that of wet spin ones. Importantly, Barus effects, that generally arise during the melt spinning of polymers, were not observed, indicating that caution must be taken when classical polymer rheology theories are applied to investigate the dynamic behaviors of GO solution.

## Introduction

Graphene has super mechanical, electrical and thermal properties, due to its sp^2^ carbon based crystal structures^1–3^. The assembly of graphene into graphene based macro-sized objects (GMO) that can inherit some outstanding characteristics of graphene is extremely important for the practical application of graphene. In the past several years, there has indeed witnessed significant advancement^4–10^. For example, Ruoff’s group firstly reported graphene oxide (GO) paper, in which graphene nanosheets are stacked tightly layer-by-layer, and these GO papers exhibited high strength and modulus^4^. Graphene aerogel/hydrogel is another typical GMO. Shi *et al*. employed hydrothermal method to synthesize graphene hydrogel, and such GMP possesses high electrical conductivity, porosity and accessible surface area, and thus provides competitive candidate for the electrodes in energy storage materials. Cheng and Ren creatively replaced the original 2-D metal catalyst foil by 3D porous one to grow graphene foam, which significantly push and trigger the research in this area^10^. All the above development manifests the versatility and great potential for GMOs.

Among all GMOs, graphene fiber (GF) is very interesting and attractive, since it is strong, lightweight, flexible and conductive, based on which a large amount of new applications might be envisioned^11–15^. Wet spinning method is a quite general strategy to prepare fibers, and in principle, any nanoparticle dispersion can be gelled into fiber when the right combination of dispersion agent and coagulation solution are employed^16,17^. Gao *et al*. took the advantage of spontaneously developed liquid crystal behaviors of GO with relatively high concentration, successfully developed a series of high performance GFs by wet-spinning^18^. Wallace *et al*. further employed DNA to wet spin synthesized GFs, aiming for the GFs based tissue engineering^19^. Interestingly, Qu *et al*. expanded the hydrothermal process to the synthesis of GFs, by using hollow glass tube as autoclave^20^. Very recently, Chen further advanced this strategy by replacing the hollow glass tube by flexible silica hollow tube, which significantly improved the GF yield^21^. The obtained graphene hybrid fibers showed excellent energy storage performance.

However, it worth to be pointed out here, that almost all the reported GFs are synthesized either by hydrothermal or wet-spin. Fiber spinning is actually a method that has been extensively used since several decades ago in textile industry, and various spinning strategies have been tested and optimized. Wet spin and dry spin are the two most used spinning method^22–24^. Comparing wet spinning method, the productivity of dry spin is much higher, and even more importantly, purification is not necessary^22^. Unfortunately, for the best of our knowledges, the studies of the dry spinning GFs are still very limited. When this paper is still under review, Gao’s group firstly reported dry spinning GFs^25^. Even though extruding high concentrated GO gel has been announced by other groups in their investigation of 3D printing graphene structures^26,27^, the rheological properties of GO, as well as its physical impact on the drying dynamics, micro-structures and mechanical properties of the prepared fibers still need to be clarified. In addition, the highly hydrophilic characteristics of GO limited the maximum concentration of GO solution we can achieve, the GO fibers immediately obtained by wet/dry spin still contain a large amount of water. This makes the drying process, during which GO sheets are assembled into closely packed structure, become the key step in order to control and modify the performance of the final GO fibers. Keeping the above considering in mind, we focused, in this study, on the dry spinnability of GO inks, and relationship between spinning conditions, including rheology properties of GO and size of spinneret, and GF mechanical properties, are established. A model for the development of microstructure during drying process of gel GOFs was also provided.

## Results and Discussion

The rate of GF synthesis by our dry spin method is very high, which is mainly determined by the spin rate. This is because comparing with wet spin, there is no need for extra treatment, such as purification, for dry spin GO fibers, which not only time consuming, but also involve chemical solvent that make potential pollution. This advantage of dry spin makes it very competitive. On the other hand, considering the GO concentration of the dry spin ink is significantly higher than that in wet spin, the behaviors of GO assembly during the spinning and drying process, as well as the micro-structure of obtained solid GO fibers could be very different. The evolvement of liquid crystal distribution as the GO ink concentration increased can be seen in Fig. S1. As the GO concentration increased from 0.5 mg/ml to 20 mg/ml, the average size of liquid crystal domain increased accordingly. However, when the concentration is as high as >40 mg/ml, the POM signals are significantly reduced because of the low transmittance of the GO solution, which prevent the qualitative measurement of the average crystal liquid size.

**Figure 1:**
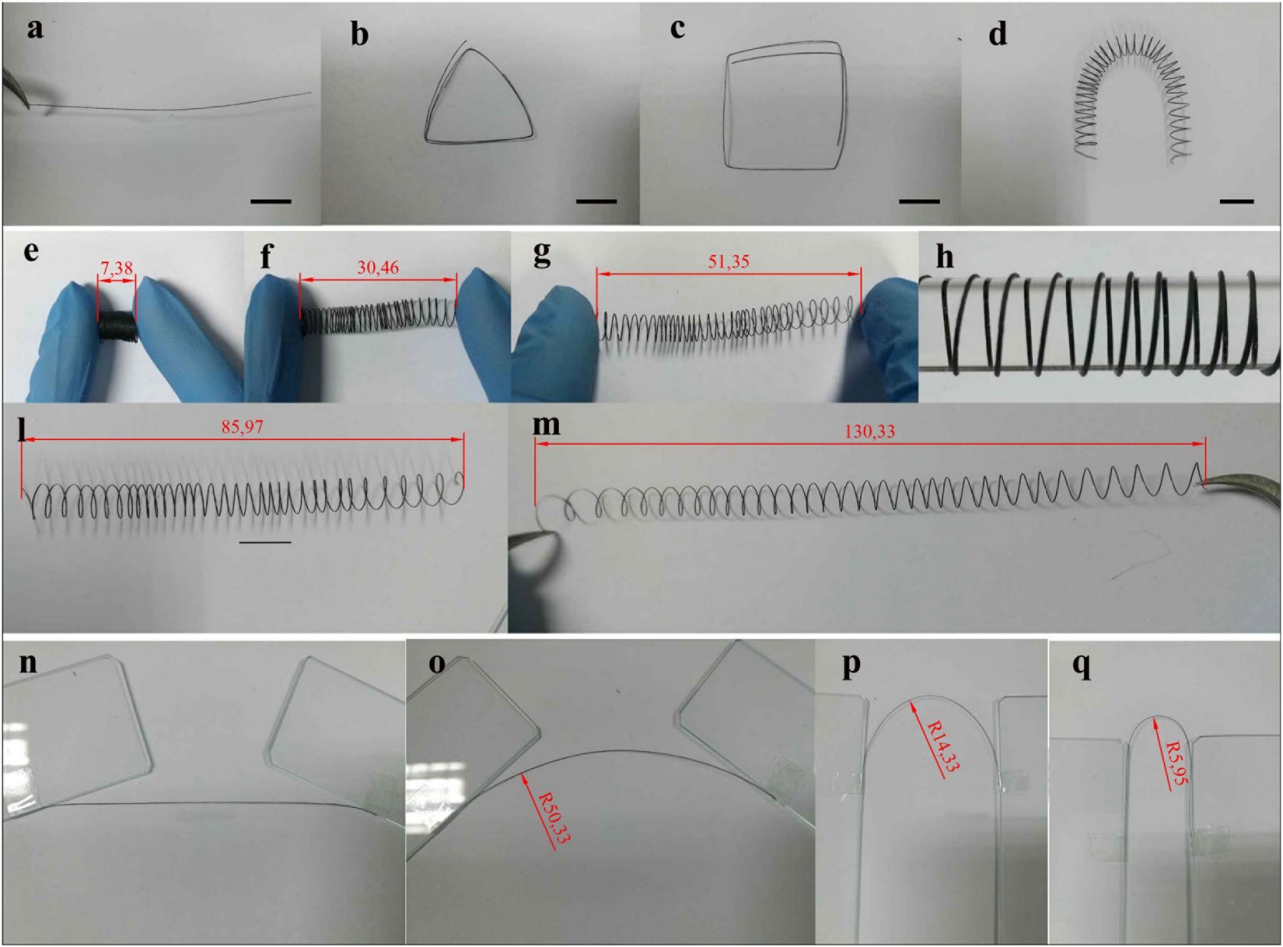
Morphology and flexibility of graphene fiber. Photographs of (**a**–**d**) GOF with various shapes; (**e**–**m**) GOF springs can be compressive and stretched to large extend. The scale bar in (**a**–**d**) is 5 mm.

The gel GOFs immediately extruded from spinneret still has a large amount of water (90–95%), however, it can still perfectly be self-supported with no observable changes of the shape during spinning process. This is guaranteed by the proper rheology properties of GO ink as will be elaborated in the following. After the GOFs were dried by fixing two ends with controlled force, it was observed that, all GFs shrink along their axial direction by ~8%, while the radial direction by ~85%. Such asymmetry shrinkage manifested that most of liquid crystal domains are aligned in the axial direction, which is induced by the shear stress generated by the spinneret during the spinning process. Because of the structure stability of the gel GOFs, they can form various complex shapes and structures shown in Fig. 1 and video in the supplementary materials. This is very different from the wet-spin process, in which fibers randomly float in coagulation solution. Such characteristic allows us to use this technique to pattern GO fibers on flexible substrate. As prove of concept, GOFs can be directed extruded on curved substrate, such as a glass bar, to fabricate a highly compressible spring shaped structure as shown in Fig. 1. This GO spring can be compressed and stretched between 7.37 mm and 130.33 mm. Considering the high chemical robustness and mechanical properties of graphene, those springs might be used to replace metal spring for vibration isolation in some chemical corrosion environment. The evolvement of microstructure of GOF during drying process was investigated by freeze-drying the GOF after dried for some time (0 min, 5 min, 10 min, 20 min, 30 min, 40 min) as shown in Fig. S2. It can be seen that, the GO nanosheets in the peripheral of the dry-spun GO fiber stacked in parallel, while packed relatively randomly in the core, thus forming core-shell structures. The thickness of the shell is mainly limited by the boundary layer. According to the fundamental fluid mechanics, the boundary layer thickness is roughly inverse proportional to the viscose of the fluid. Since the GO ink used for dry spin has very high viscosity, as compared to the case of wet spin, the boundary layer thickness is much less. This is probably one of the main reason why the dry spin GO fiber is weaker than the one obtained by wet spin as we will elaborate in the follows. In addition, the shell is formed immediately once the GO fiber is extruded out of the spinneret. This could prevent the further water evaporation and induced high capillary force, which eventually bend the shell towards the core, as can be seen in the SEM images.

**Figure 2:**
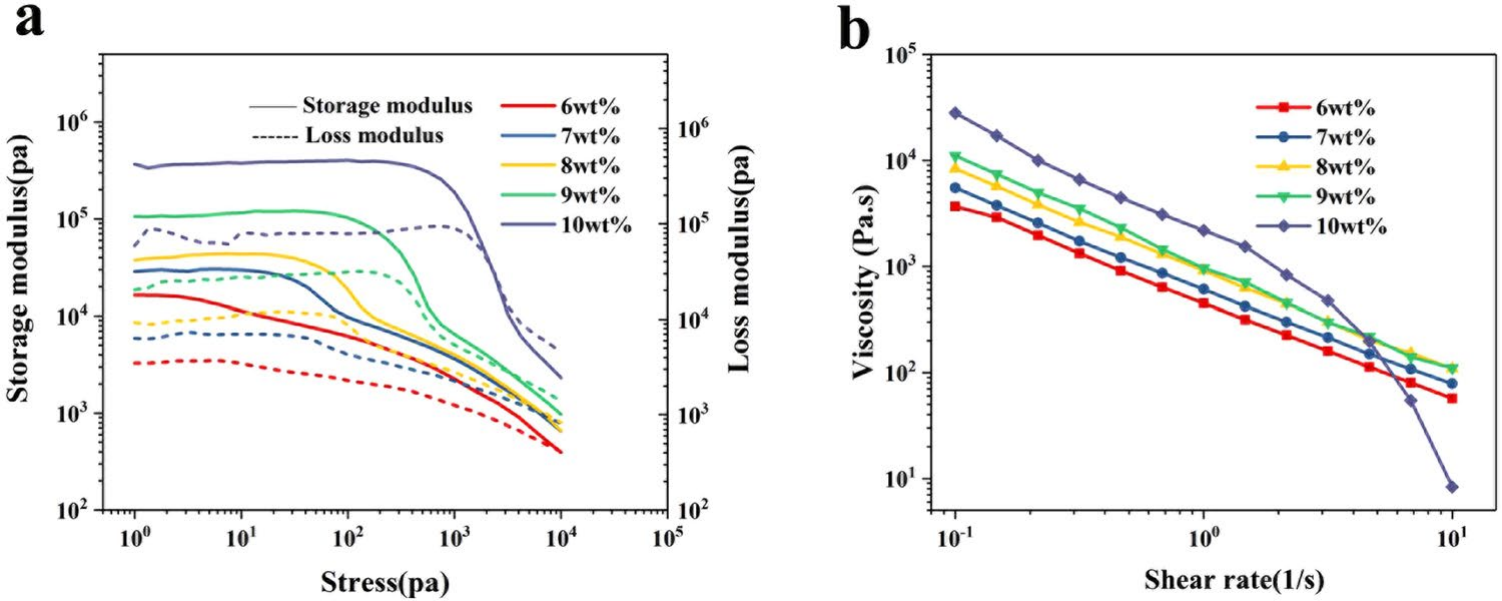
Rheology properties of the GO ink with various concentrations.

There is a critical difference between the inks for dry spin and wet spin that should be clarified, in order to elaborate the fundamental mechanisms for the micro-structure formation during drying process and related mechanical properties of GO fiber. Since coagulation agents, such as metal ions, are employed for the formation of gel GO fiber in wet-spin process, the minimum spinnable concentration of GO can be very low (less than 2 mg/ml)^12^, and thus rheology property of the GO ink is almost irrelevant. In contrast, for dry-spinning method, proper rheology performance is the key factor. It is required that once the GO ink is extruded out of the spinneret, it must have the capability to self-supported. As can be seen in Fig. 2, the rheology properties are highly dependent on the GO concentration. The storage modulus is higher than loss modulus for all the tested GO concentrations, while their difference become larger with GO concentration. For the ink with highest concentration of 10 wt%, the storage and loss modulus are as high as 0.3 MPa and 0.08 MPa, respectively. On the other hand, the yield stress, corresponding to the cross-point of storage modulus and loss modulus, gradually decreased from 9 kPa for ink of 6 wt% to 2 kPa for ink of 10 wt%. This might indicate that the average domain size of liquid crystal of GO ink increase with GO concentration, which allow GO nanosheets to align themselves more easily in a same direction under shear stress. In addition, shear thinning effects can also be clearly seen for all the tested inks (Fig. 2(b)). When the shear rate increased from 0.1 to 10 1/s, viscosity decreased significantly by more than two orders of magnitude.

**Figure 3:**
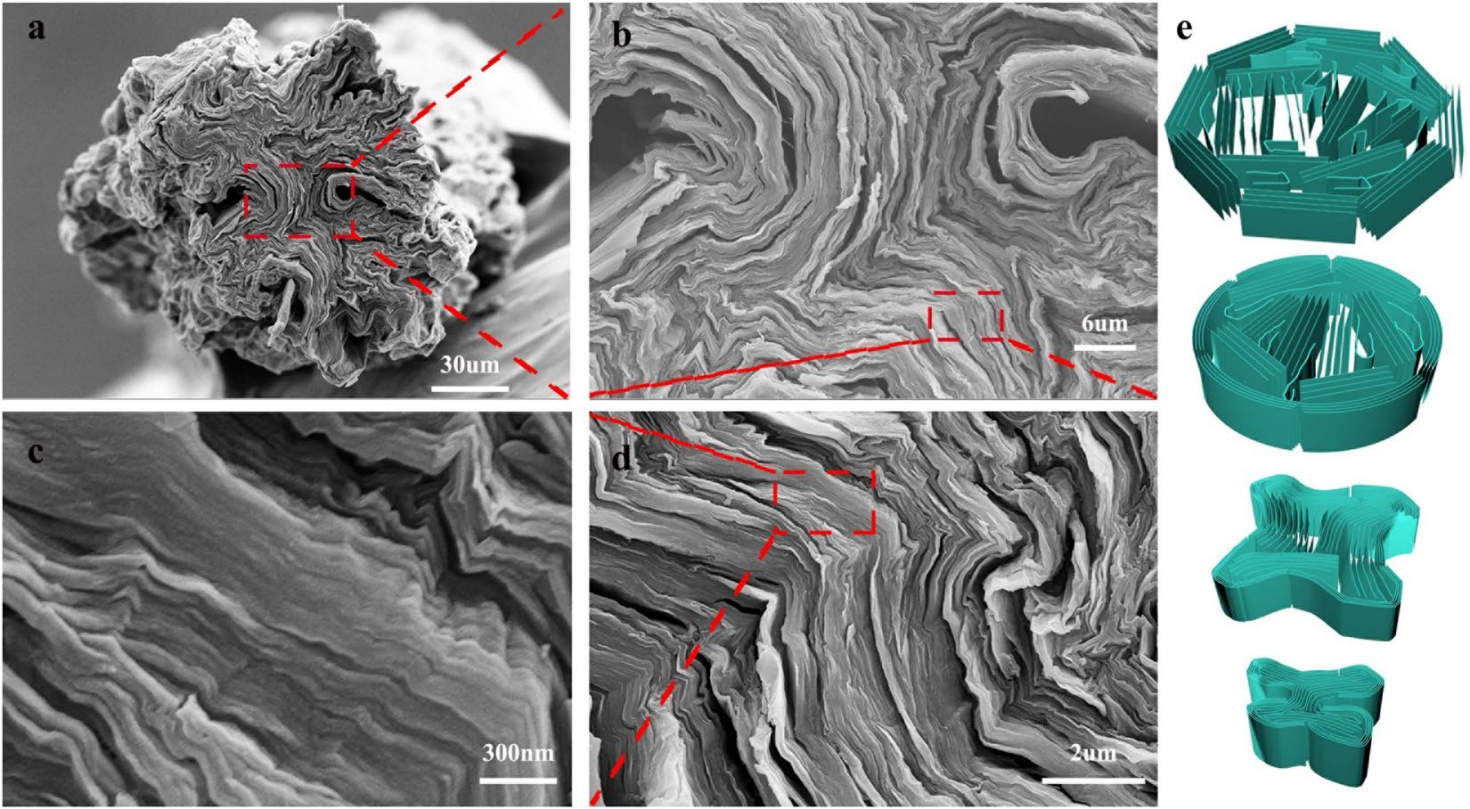
SEM images of GOFs (**a**–**d**) and schematics illustrating the GO assembly during drying process.

The micro-structures of the GO fibers are shown in Fig. 3. Interestingly, the cross-section of GOF takes a clear star shape, with the periphery forming crumples and folds towards the center of the fiber. Holes and defects due to such folding can be clearly observed, marked by the boxes in Fig. 3(b). Such morphologies are explained by analyzing the drying process of GOF as follows. It has been pointed out by many groups that, GO nanosheets close to the inner wall of spinneret are subjected to high shear stress, which align these GO nanosheets to each other and form much more and larger liquid crystal domains than that far away from the inner wall^12^. Therefore, the gel GOF immediately extruded from the spinneret has “core-shell” structure, with the shell consists of closely packed GO liquid crystal domains and core of relatively randomly distributed GO nanosheets or small GO liquid crystal domains (Fig. 3e). As water evaporated, the concentration of GO increased, the “shell” region of the gel GOF gradually evolves into water impermeable and continuous solid GO shells by connecting the original isolated GO liquid crystal domains. In fact, this is reminiscent of the strategy to synthesize GO paper at the air-liquid interface as reported by many groups. The gaps between those continuous GO shells became the only channels for water evaporation. Upon further drying, the GO shells are bent severally because of the capillary force, and many folds are generated as can be seen in Fig. 3, between which gaps and defects are naturally formed. Note that, because most of the isolated GO nanosheets are already assembled into shell or laminate structures, there is very limited number of GO nanosheets, that is flexible, are available to fill these gaps and defects. Therefore, the GOFs obtained by dry-spinning only possess relatively low mechanical properties of fibers, comparing to wet spinning. For the wet-spinning GO fibers, since the GO concentration for the ink is much lower than that of dry spinning, GO liquid crystal domains are much smaller and fewer, and a large number of GO nanosheets are still freely to be involved into the assembly process, both of which help the close compact of the whole fiber structure.

**Figure 4:**
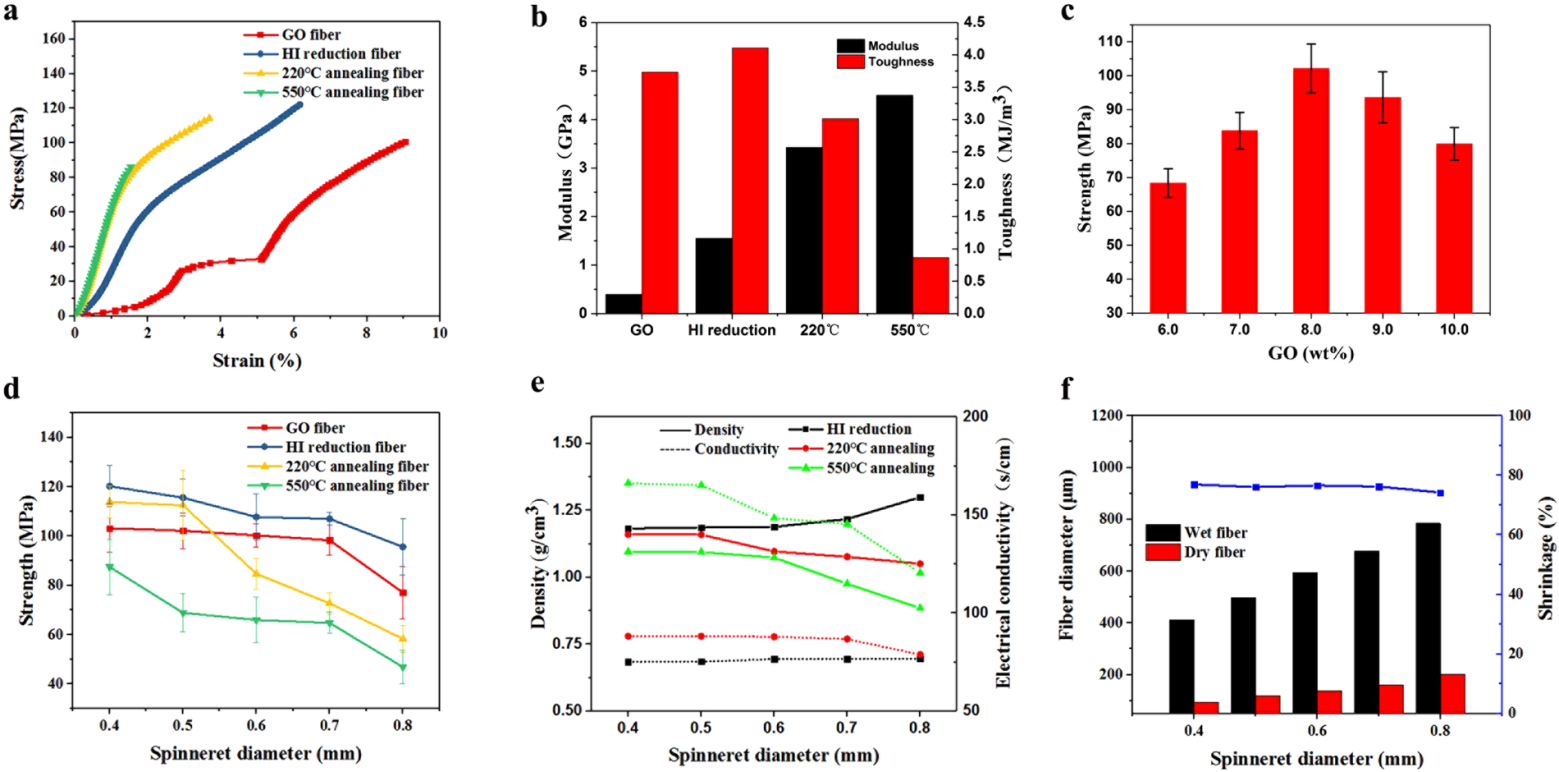
Mechanical and electrical properties. (**a**) Typical stress-strain curves for GO fibers (GOFs), HI reduced GO fibers (H_GF) and thermal treated GO fibers (HT220_GF and HT550_GF); (**b**) Modulus and toughness; (**c**) strength dependent on GO concentration; (**d**) strength of the fibers at before and after reduction; (**e**) The dependence of electrical conductivity and density on spinneret diameter. (**f**) Barus effects investigation.

As the initial study for the dry spinning of GOFs, we mainly focused on the effects of GO concentration, diameter of spinneret and chemical reduction conditions on the mechanical and electrical properties of GFs. Typical stress-strain curves are showed in Fig. 4(a). For GOFs, the stress increase with strain with a strengthen stage between 2% and 3%, followed by a “yield” stage in the strain range of 3% and 5%. Upon further stretching, a second strengthen stage immerge. Such evolvement of stress-strain curve is similar to that of the foam structure with hierarchical architectures, and can be explained by the micro-structures of GOFs as elaborated above. More specifically, the defects and gaps formed spontaneously during the drying process can result to the slippery between different shells under tensile stress. Due to the Possion’s ratio effect, when the fiber is further stretched along axial direction, the gaps are closed gradually by compressive strain in the plane of cross-section, which activates internal static frictions and thus effectively increase the stiffness of the GOF. This can explain the observation that Young’s modulus of GOF is increased when the strain is less than 3%. However, when external stress exceeds some critical threshold value, graphene shells/laminates start to slip with each other, corresponding to the “yield” stage between 3% to 5%, until these graphene shells tightly compact once again and thus strengthen the whole GOF structures. The fracture strain of GOF is as high as 9%, and much higher than the counterpart of wet spin GOF^12^, and is comparable with the ones that scrolled from GO film^28^. The GOF has an average tensile strength of 105 MPa, Young’s modulus of 0.4 GPa, and fracture toughness of 5 MJ/m^3^, which is similar to structural nature materials, such as nacre. Upon chemical reduction by HI, the Young’s modulus, strength and toughness are all increased, with the former much more significantly (increased by ~200%). Thermal treatment (at 220 °C) can further enhance the modulus but not the strength, and also decrease the fracture strain significantly. “Yield” stage is not observed for the reduced samples. Indeed, as reported by Pei, there would be significant amount of iodine laminated between GO layers, which might reinforce the whole structures^29^. Therefore, the gaps and defects in GOFs are probably filled at least partially with the residue iodine, and thus prevent the slippery of GO shells relative to each other. After annealing at 550 °C for two hours, at which most of iodine residue is volatilized to gas, the strength even decreased, and this can be related to the inevitable gas generated porosity. We microstructure and chemical composition of GOF after different reduction treatment was also investigated by SEM and XPS, respectively (Figs S3–4). It can be seen that, after HI reduction, the structure of GO fiber became much fluffier, while the core-shell structure is more or less maintained. The thermal treatment at 220 °C and 550 °C do not change the morphology significantly, relatively intact shell structure can be distinguished. Interestingly, based on XPS data, the GO fiber is only partially reduced, with the extent much less than what we expected. This is probably because of the dense and parallel packing of GO nanosheets in the shell of the fiber. As illustrated by Geim’s group, reduced GO film is impenetrable for all molecules, even Helium^30,31^. The shell structure prevents the diffusion of HI agent deep into GO fibers, and thus greatly limits the reduction of GO. As for the case of thermal treatment, the reduction process could also be inhibited by such confinement.

The GO ink concentration and spinneret diameter dependent of the strength were also investigated. Figure 4(c) shows that the maximum strength is obtained for the fiber with concentration of 8%. This is probably related to the fact that clogs of spinneret occur now and then when GO is very high (>8%). Indeed, when the average domain size of GO liquid crystal is comparable to the diameter of spinneret, the smooth extrusion of GO ink could be interrupted (Fig. S1). On the other hand, with increasing the spinneret diameter (Fig. 4(d)), the strength of all fibers decrease, which is mainly due to the less alignment of GO that induced by larger spinneret diameter. Figure 4(e) also shows the dependence of density and electrical conductivity of GFs on the spinneret diameter. Interestingly, it seems that both the density and conductivity are insensitive to the spinneret diameter, except for the HT550_GFs. The conductivities of H_GF, HT220_GF and HT550_GF are 75, 85 and 112 S/cm, respectively, which is comparable to the GFs obtained from wet-spin. This should be related to the fact that most of the iodine residues in fibers can be eliminated by thermal treatment at 550 °C, which results to pores and gaps inside GFs. Further, since there is no obvious chemical structural difference among H_GF, HT220_GF and HT550_GF, the enhancement of conductivity should be mainly correlated with the increase of density. In addition, the density of our GFs are actually much higher that of solid wet-spin fibers^32^. This indicates that although pores and gaps exist in our fibers, the average compaction of graphene is still much denser than that of wet spin graphene fibers. Note that it has been concluded by Gao, larger domain of GO liquid crystal results to higher strength. However, we did not observe such pattern. This is because when the domains of GO liquid crystal are too large, they can assembly into rigid shells and limit the close compaction of the whole structure afterward as we have discussed above. Therefore, a complete picture of GO assembly during drying process must be established, and more specifically, flexibility of assembly units should be maintained before the drying out of the gel GOFs.

It is well known that Barus effects (expansion of fibers) cannot be avoided during the polymer fiber extrusion^33,34^. Considering the similarity of structures between GO and polymers in terms of characteristic length scale and flexibility, we initially expected Barus effects can be observed for dry spinning GOF. However, the diameters of dry spin GO fiber is almost equal to that of spinneret, without any noticeable diameter expansion effects. The mechanism for the Barus effects of polymer fiber extrusion is the recovery of elastic deformation of polymer chains once the fiber is extruded out of the spinneret. GO nanosheets has much higher in-plan rigidity than that of the flexible polymer chains, and it seems impossible to deform GO by hydrostatic force in the in-plan directions. In addition, since the bending stiffness of GO nanosheets is negligible, the elastic energy storage by bending of GO seems impractical^34^. Therefore, it is worth pointing out that the rheology behaviors of GO ink cannot be directly explained by the polymer dynamic theories. We envisioned that the shear thinning effect of GO inks is intimately related to the super-hydrophilic properties of GO, and it is the modification of dynamics of water molecules by GO that should account for most of the rheology performance of GO ink.

## Conclusion

In summary, we reported the synthesis of graphene fiber via dry-spinning method. The graphene fibers exhibited a strength of ~120 MPa, comparative to the firstly reported wet-spin fibers by Gao’s group, and high fracture strain of 9%, which is much higher than that of wet-spin GO fiber^12^. Such difference is probably caused by the much larger and thicker domains of GO liquid crystal than that in ink for wet-spinning, which constrain the dense packaging of GO nanosheets and leave behind pores and gaps that eventually limit the strength of GO fibers. However, direct evidence for such conjecture still requires *in-situ* experiments. The comparison between the drying process for wet spin GO fiber and dry spin GO fiber also indicates both the GO liquid crystal domain size and availability of flexible GO nanosheets should be taken into consideration for the synthesis of high strong and conductive graphene fibers. The Barus effects were not observed for the dry spinning of GOFs, which suggests that it should be cautious when polymer rheology theories are applied to study the colloidal behaviors of GO solutions.

## Experimental

### Materials

Graphene oxide was synthesized from purified natural graphite by the modified Hummers method. The size distribution can be seen in Fig. S4. The GO dispersion was placed in Teflon breaker and heated in a water bath at 50 °C with continuously stirring by a Teflon rod to obtain a highly concentrated graphene oxide in a gel form. Inks with concentrations of 6 wt%, 7 wt%, 8 wt%, 9 wt% and 10 wt%, were prepared.

### Dry spin GO fibers and characterization

The graphene oxide fiber (GOF) was made by printing the highly concentrated GO use a dispenser (Ultimus V High Precision Dispenser, Nordson), followed by air drying with both ends of the flexible fixed. The fiber diameter just spun out was measured using Transmission polarized light microscope (MP41, Mshot). The GOFs were reduced by HI with concentration of 20 wt% at 80 °C for 10 h, and then washed by distilled water for three times, and the obtained fibers are termed as H_GFs. To prepare the annealing samples, the H_GFs were annealed at temperature of 220 °C or 550 °C, in a tube furnace oven (TF55030C-1, Lindberg/Blue) under the protection of argon. The thermal reduced fibers are termed as HT220_GF and HT550_GF, with the number indicating annealing temperatures. If it is not clearly pointed out, the fibers are fabricated by using ink with the concentration of 8 wt% and spinneret with diameter of 0.4 mm, since it can result to the best mechanical properties as will be discussed. The rheology and viscosity of the GO solution were measured using a discover HR-2 rheometer. The fiber static tensile tests were conducted with a dynamic mechanical analyzer (DMA Q800, TA Instruments). The sample was glued to the paper for mechanical testing and was gripped using a film tension clamp. All tensile tests were conducted in a controlled force rate mode with a force ramp rate of 0.5 Nmin^−1^. Conductivity measurements were carried out on a multi-meter. SEM images were obtained BY using a supra 55 sapphire operated at an accelerating voltage of 10 KV.

## Electronic supplementary material

Supplementary Information

## References

[1] Geim AK, Novoselov KS (2007). The rise of graphene. Nature Material..

[2] Stankovich S (2006). Graphene-based composite materials. Nature..

[3] Lee C, Wei X, Kysar JW, Hone J (2008). Measurement of the elastic properties and intrinsic strength of monolayer graphene. Science..

[4] Dikin DA (2007). Preparation and characterization of graphene oxide paper. Nature..

[5] Hu H, Zhao Z, Wan W, Gogotsi Y, Qiu J (2013). Ultralight and highly compressible graphene aerogels. Advanced Materials..

[6] Zhu C (2015). Highly compressible 3D periodic graphene aerogel microlattices. Nature Communications..

[7] Chen Z (2011). Three-dimensional flexible and conductive interconnected graphene networks grown by chemical vapour deposition. Nature Materials..

[8] Chen Z, Xu C, Ma C, Ren W, Cheng H (2013). Lightweight and flexible graphene foam composites for high‐performance electromagnetic interference shielding. Advanced Materials..

[9] Chen H, Müller MB, Gilmore KJ, Wallace GG, Li D (2008). Mechanically strong, electrically conductive, and biocompatible graphene paper. Advanced Materials..

[10] Yang X, Cheng C, Wang Y, Qiu L, Li D (2013). Liquid-mediated dense integration of graphene materials for compact capacitive energy storage. Science..

[11] Meng F (2015). Graphene-based fibers: a review. Advanced Materials..

[12] Xu Z, Gao C (2014). Graphene in macroscopic order: liquid crystals and wet-spun fibers. Accounts of Chemical Research..

[13] Cheng H, Hu C, Zhao Y, Qu L (2014). Graphene fiber: a new material platform for unique applications. NPG Asia Materials..

[14] Cao X, Yin Z, Zhang H (2014). Three-dimensional graphene materials: preparation, structures and application in supercapacitors. Energy & Environmental Science..

[15] Cong HP, Chen JF, Yu SH (2014). Graphene-based macroscopic assemblies and architectures: an emerging material system. Chemical Society Reviews..

[16] Liu YJ, Xu Z, Gao WW, Cheng ZD, Gao C (2017). Graphene and other 2D colloids: liquid crystals and macroscopic fibers. Advanced Materials..

[17] Bai XP (2017). Continuous draw spinning of extra-long silver submicron fibers with micrometer patterning capability. Nano Letter..

[18] Xu Z, Sun H, Zhao X, Gao C (2013). Ultrastrong fibers assembled from giant graphene oxide sheets. Advanced Materials..

[19] Aboutalebi SH (2014). High-performance multifunctional graphene Yarns: toward wearable all-carbon energy storage textiles. ACS Nano..

[20] Dong Z (2012). Facile fabrication of light, flexible and multifunctional graphene fibers. Advanced Materials..

[21] Yu D (2014). Scalable synthesis of hierarchically structured carbon nanotube-graphene fibres for capacitive energy storage. Nature Nanotechnology..

[22] Walczak ZK (2002). Process of fiber formation. Elsevier Science..

[23] Dzenis Y (2004). Spinning continuous fibers for nanotechnology. Science..

[24] White JL (1964). Dynamics of viscoelastic fluids, melt fracture, and the rheology of fiber spinning. Journal of Applied Polymer Science..

[25] Tian QS (2017). Drying spinning approach to continuous graphene fibers with high toughness. Nano scale..

[26] Kim JH (2015). 3D printing of reduced graphene oxide nanowires. Advanced Materials..

[27] Zhu C (2015). Nature Communications..

[28] Cruz-Silva R (2014). Super-stretchable graphene oxide macroscopic fibers with outstanding knotability fabricated by dry film scrolling. ACS Nano..

[29] Pei S, Cheng HM (2012). The reduction of graphene oxide. Carbon..

[30] Su Y (2014). Impermeable barrier films and protective coatings based on reduced graphene oxide. Nature Communications..

[31] Joshi RK (2014). Precise and ultrafast molecular sieving through graphene oxide membranes. Science..

[32] Chen L (2013). Toward high performance graphene fibers. Nanoscale..

[33] Newman S, Trementozzi QA (1965). Barus effect in filled polymer melts. Journal of Applied Polymer Science..

[34] Bagley EB, DUffey HJ (1979). Recoverable shear strain and Barus effect in polymer extrusion. Transactions of the Society of Rheology..

