# Supplementary material for: Dry Spin Graphene Oxide Fibers: Mechanical/Electrical Properties and Microstructure Evolution
Source: Sci Rep. 2018 Jul 17;8:10803. doi: 10.1038/s41598-018-29157-4 (PMC6050277; doi:10.1038/s41598-018-29157-4)
Supplement: Supplementary file 1 — Supplementary Information [file 41598_2018_29157_MOESM1_ESM.docx]

**Dry Spin Graphene Oxide Fibers: Mechanical/Electrical Properties and Microstructure Evolution**

Lichao Feng^a,b,c^, Ying Chang^a,b,c^, Jing Zhong^b,c,d,*^, De-Chang Jia^d,e,*^

*^a^School of Mechanical Engineering, Huaihai Institute of Technology, and Marine Resources Development Institute of Jiangsu, Lianyungang 222005, Jiangsu, China*

*^b^Key Lab of Structure Dynamic Behavior and Control (Harbin Institute of Technology), Ministry of Education, Harbin 150090, Heilongjiang, China.*

*^c^School of Civil Engineering, Harbin Institute of Technology, Harbin 150090, China.*

*^d^Institute for Advanced Ceramics, Harbin Institute of Technology, Harbin 150080, China*

*^e^School of Materials Science and Engineering, Harbin Institute of Technology, Harbin 150010, China.*

*Email:* [zhongjing@hit.edu.cn](mailto:zhongjing@hit.edu.cn); dcjia@hit.edu.cn

Figure S1. POM images of graphene oxide with different concentrations. (a) 0.5 mg/ml, (b) 5 mg/ml, (c) 20 mg/ml, (d) 40 mg/ml, (e) 60 mg/ml and (f) 100 mg/ml.





Figure S2. The evolvement of microstructure of GOF during drying process with elapse of time after fiber extrusion. (a) 0 min (b) 5 min (c) 10 min (d) 20 min (e) 30 min (f) 40 min.





Figure S3 (a,d) Treated by HI agent, (b,e) treated by HI agent and annealing at 220 oC, (c,f) treated by HI agent and annealing at 550 oC.


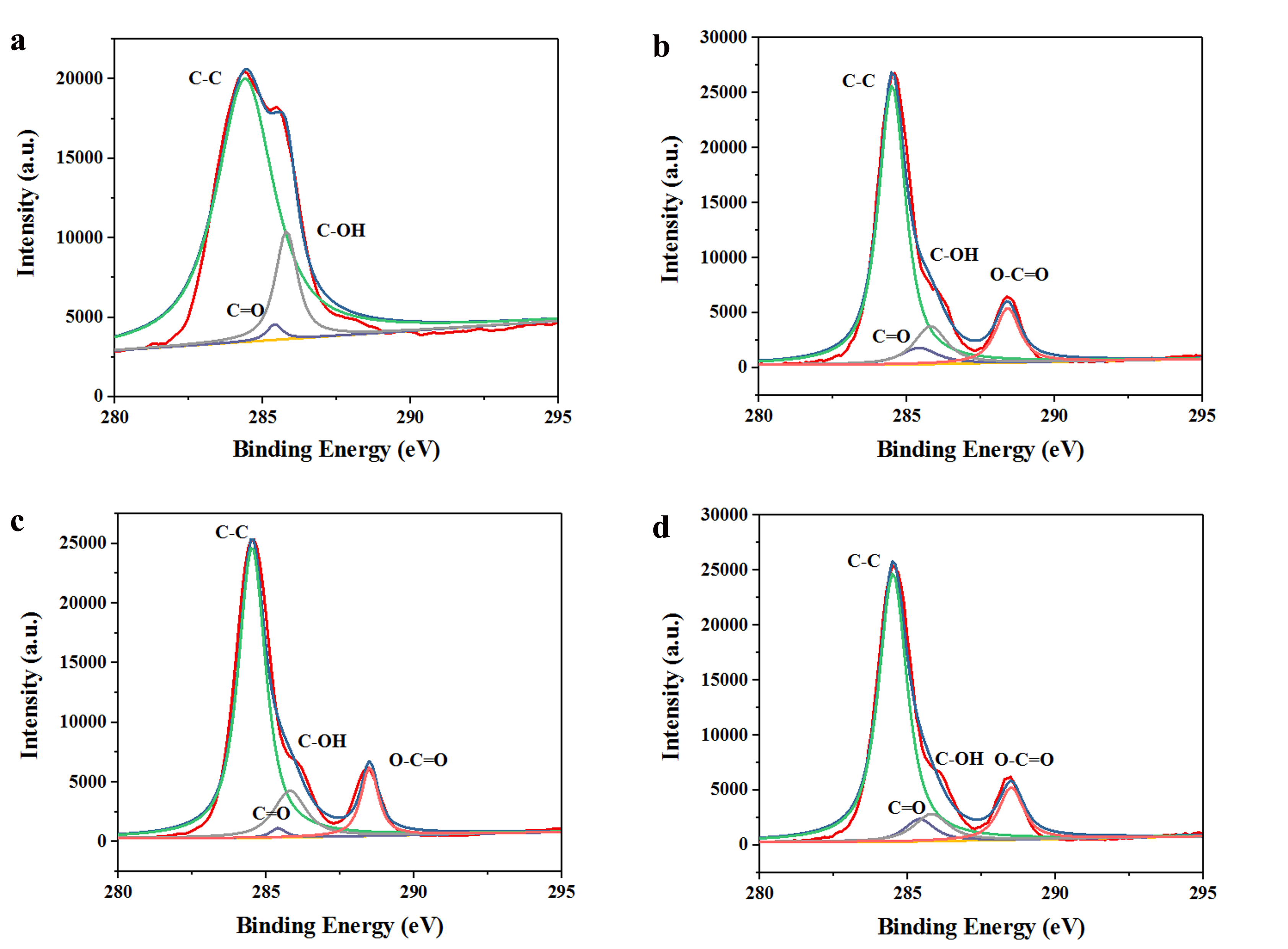


Figure S4. XPS data for GOF (a), GOF reated by HI agent (b), GOF treated by HI agent and annealing at 220 oC (c), GOF treated by HI agent and annealing at 550 oC (d).





Figure S5. Size distribution of GO nanosheets.


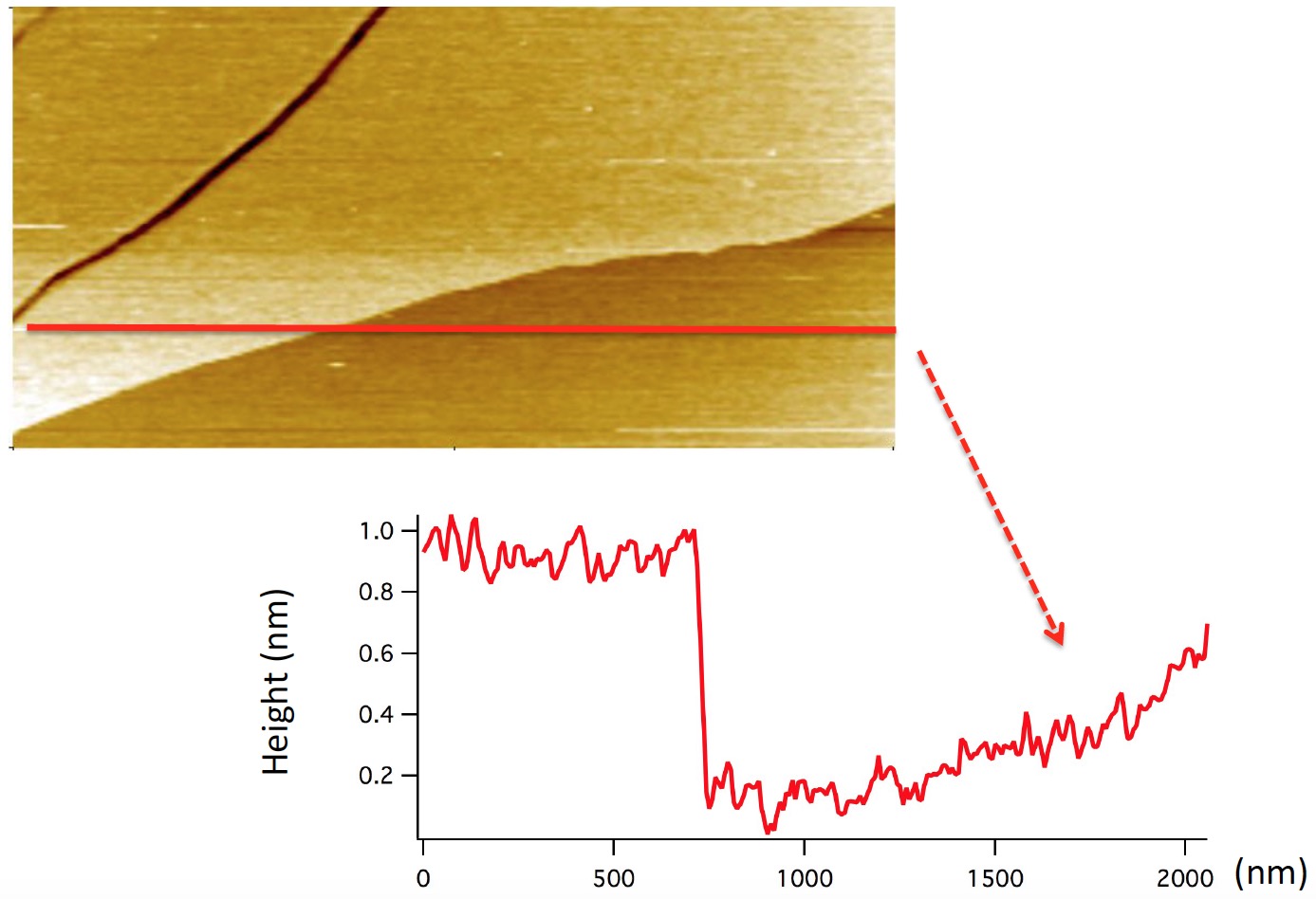

Figure S6. A height profile taken by AFM on the edge of a flake indicate a thickness of about 0.8-1.0 nm.
